# Illness Perceptions in Psychiatric Disorders: Assessing Differences and Associations With Symptom Severity

**DOI:** 10.1002/erv.70036

**Published:** 2025-10-08

**Authors:** Bede Carr, An Binh Dang, Litza Kiropoulos, Isabel Krug

**Affiliations:** ^1^ Department of Psychiatry St Vincent's Hospital Melbourne Melbourne Australia; ^2^ Melbourne School of Psychological Sciences University of Melbourne Melbourne Australia

**Keywords:** Anorexia Nervosa, Brief Illness Perception Questionnaire, Generalized Anxiety Disorder, illness perceptions, Major Depressive Disorder, mental health, Panic Disorder, Self‐Regulatory Model, Social Anxiety Disorder

## Abstract

**Objectives:**

This study investigated the influence of illness perceptions (IPs) on clinical severity across Major Depressive Disorder (MDD), Anorexia Nervosa (AN), Generalised Anxiety Disorder (GAD), Social Anxiety Disorder (SAD), and Panic Disorder (PD), and compared the IPs across these conditions.

**Method:**

We administered the Brief Illness Perception Questionnaire alongside disorder‐specific severity scales to 774 participants (85.1% female).

**Results:**

Significant associations between IPs and symptom severity emerged for AN and GAD only. Higher perceived Personal Control was associated with reduced severity in GAD but with greater severity in AN, whereas higher perceived Treatment Control was linked to lower severity in AN. In contrast, stronger perceptions of Concern were associated with increased severity in GAD. Although several IP dimensions demonstrated transdiagnostic overlap, differential associations also emerged, particularly for AN with respect to Timeline, Identity, and Emotional Representations.

**Discussion:**

Our findings underscore the critical role of IPs in mental health severity and support the need for tailored recovery‐oriented interventions targeting specific maladaptive IPs to improve clinical outcomes. However, given the high prevalence of comorbidity across the assessed disorders, the findings should be interpreted with caution, as overlapping conditions may have shaped both IPs and their associations with severity.

## Highlights

- Illness perceptions (IPs) were assessed across Major Depressive Disorder (MDD), Anorexia Nervosa (AN), Generalised Anxiety Disorder (GAD), Social Anxiety Disorder (SAD), and Panic Disorder (PD).
- Significant IP severity links were found only for AN and GAD.
- Control beliefs were strongly tied to higher severity in AN, but to lower severity in GAD; Concern amplified severity in GAD.
- Despite transdiagnostic overlap, AN showed distinct differences in Timeline, Identity, and Emotional Representations.
- High comorbidity may have influenced IPs and their associations with severity.

## 1 Introduction

Mental health problems defined by symptomatology consistent with conditions such as Major Depressive Disorder (MDD) (Kessler and Bromet 2013), Anorexia Nervosa (AN) (Smink et al. 2012; Averous et al. 2021), Generalized Anxiety Disorder (GAD) (Bandelow and Michaelis 2015; Penninx et al. 2021), Social Anxiety Disorder (SAD) (Stein et al. 2017), and Panic Disorder (PD) (De Jonge et al. 2016), present profound clinical challenges. They encompass distorted cognitions, emotional burdens, and sometimes life‐threatening consequences. The burden of these conditions extends across various domains, including high levels of morbidity and disability (Vigo et al. 2016), increased rates of mortality (Plana‐Ripoll et al. 2019; Krug et al. 2025), financial strain (Frankham et al. 2020), and significant disruptions to educational and occupational opportunities (McLeod and Fettes 2007).

Understanding how individuals perceive and interpret their illnesses is crucial for managing these mental health conditions effectively. The Brief Illness Perception Questionnaire (BIPQ) (Broadbent et al. 2006) offers a concise psychometrically sound framework to capture individuals' perceptions of their mental health conditions. Rooted in the Self‐Regulatory Model (SRM) (Leventhal et al. 1980, 1984; Baines and Wittkowski 2013), the BIPQ encapsulates key dimensions of illness perceptions (IPs): Identity, Cause, Timeline, Consequences, Controllability, Coherence and Emotional Representations. Although using a single item per dimension is less comprehensive than the 38‐item Illness Perception Questionnaire‐Revised (IPQ‐R; Moss‐Morris et al. 2002), the BIPQ's concise 8‐item format enables rapid assessment in clinical settings. Each dimension is rated on a 0–10 scale, providing an efficient way to capture patients' cognitive and emotional representations and guide targeted interventions (Broadbent et al. 2006).

While the BIPQ has been extensively used to examine IPs in non‐psychiatric disorders (Broadbent et al. 2015), its application in mental health conditions remains limited. Research has mainly focused on its relationship with psychosocial adjustment and adherence to treatment in individuals with MDD (Averous et al. 2021; Broadbent et al. 2006; Vervoort et al. 2016; Glattacker et al. 2013; Spain et al. 2007), while neglecting other disorders that are frequently comorbid with MDD, such as anxiety disorders (Ter Meulen et al. 2021) and eating disorders (Hambleton et al. 2022). The current study addresses this gap by examining IPs in MDD, eating disorders, and anxiety disorders, enabling a broader understanding of IPs across highly comorbid and often overlapping mental health conditions.

### 1.1 Prior Studies on Illness Perceptions Across Mental Health Disorders

#### 1.1.1 Major Depressive Disorder (MDD)

The SRM has been validated as a reliable framework for understanding IPs in individuals with depression (Fortune et al. 2004), with findings in MDD indicating that, although these perceptions may not directly relate to symptom severity, they are strongly associated with treatment‐related behaviours. Accordingly, more negative illness views have been linked to lower help‐seeking, poorer treatment adherence, and reduced engagement with preferred interventions (Manber et al. 2003; Munson et al. 2009). However, the relationship between specific IP dimensions and depressive symptom severity remains underexplored, representing a notable gap in the literature.

#### 1.1.2 Anorexia Nervosa

In research involving individuals with AN, studies have consistently shown that IPs are closely linked to clinical eating disorder symptom severity. Early work found that people with AN often view their illness as chronic and distressing, with serious consequences, little sense of personal control, and low confidence in the effectiveness of treatment (Holliday et al. 2005; Stockford et al. 2007). These maladaptive perceptions, particularly viewing the condition as having a long timeline and being outside of one's control, are reliably linked to a greater eating disorder symptom severity (Stockford et al. 2007; Agüera et al. 2021).

Highlighting the complexities of AN, some evidence reveals a potential paradox; when compared directly with other eating disorders (Bulimia Nervosa, Binge Eating Disorder [BED] and Other Specified Feeding or Eating Disorder [OSFED]), individuals with AN reported their condition as less chronic and with fewer negative consequences. This finding may reflect the poor insight or the ego‐syntonic nature of the condition, wherein the illness is deeply integrated into a person’s identity and sense of self (Agüera et al., 2021). Such complexities therefore underscore the need for nuanced assessment and tailored interventions that address both the cognitive representations of the illness and the unique motivational barriers to change in AN.

#### 1.1.3 Anxiety Disorders

Research exploring IPs within anxiety disorders reveals a complex landscape, highlighting not only key differences between conditions but also direct associations with clinical severity. When comparing across different anxiety conditions, individuals with symptoms of PD have been found to report lower personal and treatment control around their symptoms than those with SAD, a specific distinction that holds even when accounting for underlying anxiety sensitivity (Dias et al. 2018).

In primary care settings for GAD, perceptions of severe consequences, low personal control, poor illness coherence, and biological causal beliefs have been linked to higher anxiety levels (Yap et al. 2019). In PD, El Amiri et al. (2018) examined the role of causal attributions in receiving cognitive behavioural therapy. They found that biological beliefs were associated with sex, illness duration and family history of psychiatric illness, while environmental beliefs were related to prior psychotherapy. Both types of beliefs predicted symptom severity 12 weeks after treatment. Together, these findings suggest that targeting specific IPs could be a promising avenue for improving treatment outcomes in anxiety disorders.

### 1.2 Addressing Literature Gaps Through a Transdiagnostic and Recovery‐Focused Lens

Despite advances in understanding IPs, most existing research has been conducted in isolation for specific disorders, often with small sample sizes that limit robustness and generalisability. As a result, it remains unclear whether IPs differ meaningfully across mental health conditions. Moreover, confounding variables such as gender and age are rarely controlled for, potentially influencing findings. Adopting a transdiagnostic perspective can bridge the gap between research confined to diagnostic categories and the realities of clinical practice, where comorbidity is common (Dalgleish et al. 2020). Such an approach also facilitates the identification of shared and disorder‐specific processes, enabling more targeted, efficient, and personalised interventions.

IPs in mental disorders are also relevant to the recovery model in psychiatry, which emphasises patient involvement, empowerment, and a holistic, person‐centred approach (Jacobson and Greenley 2001; Slade et al. 2008). By understanding patients' IPs, clinicians can tailor interventions that align with their perspectives, enhancing treatment adherence, satisfaction, and outcomes (Leamy et al. 2011). Integrating IPs into shared decision‐making fosters a sense of ownership and control—core principles of recovery (Anthony 1993)—and supports not only clinical goals of symptom reduction and functional improvement, but also personal goals of living a fulfilling, meaningful life despite mental health challenges (Davidson et al. 2005; Leamy et al. 2011; Ramon et al. 2007).

### 1.3 The Current Study

The present study focused on MDD, AN, GAD, SAD and PD, which are highly comorbid (McGrath et al. 2020) and share overlapping cognitive (Arango et al. 2021), emotional (Hogg et al. 2023), and neurobiological (Meyer‐Lindenberg and Tost 2012) risk factors. By assessing IPs concurrently across these conditions, the study enabled direct comparison of how different mental health disorders influence these perceptions, addressing limitations of prior research that examined disorders in isolation. Additional mental health diagnostic categories were not included due to practical constraints and to minimise participant burden, ensuring a focused and feasible scope. Understanding both shared and disorder‐specific IPs is particularly important given the diagnostic uncertainty, overlap, and comorbidity common in mental health (Aftab and Ryznar 2021).

Accordingly, this study aimed to investigate the influence of IPs on clinical severity across MDD, AN, GAD, SAD and PD. By utilizing the well‐validated BIPQ, the study aimed to uncover how individuals perceive their illnesses across various IP dimensions and if these perceptions impacted their clinical presentations. Additionally, the study aimed to explore IPs cross‐diagnostically to identify commonalities and differences in IPs among individuals with different mental health disorders.

It was hypothesised that: more negative IPs would be associated with increased clinical severity across MDD, AN, GAD, SAD, and PD (H1) and that IPs would show both shared features across disorders and disorder‐specific patterns (H2).

## 2 Methods

### 2.1 Participants

A total of 774 participants were included, categorized into five diagnostic groups: MDD, AN, GAD, SAD and PD. Demographic information, self‐reported in nature, summarized in Table 1, revealed a predominantly female sample (86.7%), with participants ranging in age from 18 to 67 years. Significant differences were found in gender distribution, sexual orientation, education level, and ethnicity. While these differences were statistically significant, effect sizes were generally small, indicating that the magnitude of group‐level demographic variation was modest. The sample exhibited high levels of comorbidity, with significant overlap between diagnostic groups, as detailed in Table 2. Despite these variations, the overall sample offered a broad demographic range, facilitating an examination of IPs across diverse mental health presentations.

**TABLE 1:** Participant demographics across diagnostic groups.

	MDD (*n* = 152)		SAD (*n* = 126)		GAD (*n* = 297)		PD (*n* = 62)		AN (*n* = 125)		*p*
	M	SD	M	SD	M	SD	M	SD	M	SD	
Age	27.95	9.52	27.54	8.74	28.65	11.05	29.82	10.76	28.06	8.61	0.596
BMI	26.08^a^	8.62	24.69^b^	8.16	26.4^c^	8.05	28.01^b,d^	10.26	21.77^a,c,d^	5.36	< 0.001

	*n*	%	*n*	%	*n*	%	*n*	%	*n*	%	*p*
Gender											0.003
Male	13	8.55	16	12.7	22	7.41	5	8.06	24	19.2	
Female	130	85.53	107	84.92	266	89.56	57	91.94	99	79.2	
Other	9	5.92	3	2.38	9	3.03	0	0	2	1.6	
Sexual orientation											0.105
Heterosexual	78	51.32	73	57.94	183	61.62	39	62.9	89	71.2	
Homosexual	10	6.58	8	6.35	13	4.38	3	4.84	9	7.2	
Bisexual	44	28.95	28	22.22	62	20.88	11	17.74	17	13.6	
Other	8	5.26	8	6.35	16	5.39	4	6.45	8	6.4	
Asexual	4	2.63	4	3.17	10	3.37	3	4.84	2	1.6	
Pansexual	8	5.26	5	3.97	13	4.38	2	3.23	0	0	
Education											0.393
Secondary school	1	0.66	4	3.17	7	2.36	3	4.84	1	0.8	
Year 12 or equivalent	55	36.18	38	30.16	125	42.09	17	27.42	38	30.4	
Certificate level	15	9.87	13	10.32	26	8.75	9	14.52	11	8.8	
Graduate diploma/certificate	2	1.32	4	3.17	5	1.68	2	3.23	9	7.2	
Advanced diploma	9	5.92	16	12.7	23	7.74	7	11.29	17	13.6	
Bachelor's degree	39	25.66	38	30.16	62	20.88	20	32.26	37	29.6	
Postgraduate degree	31	20.39	13	10.32	49	16.5	4	6.45	12	9.6	
Ethnicity											0.598
African American	7	4.61	7	5.56	8	2.69	5	8.06	13	10.4	
Caucasian	117	76.97	84	66.67	227	76.43	44	70.97	87	69.6	
Eastern Asian	7	4.61	9	7.14	14	4.71	3	4.84	2	1.6	
Southern Asian	7	4.61	6	4.76	14	4.71	4	6.45	4	3.2	
Latin American	3	1.97	2	1.59	6	2.02	2	3.23	3	2.4	
Middle Eastern	0	0	6	4.76	1	0.34	0	0	6	4.8	
Native Hawaiian or other Pacific Islander	0	0	1	0.79	0	0	0	0	1	0.8	
Indigenous Australian and/or Torres strait Islander	9	5.92	8	6.35	19	6.4	4	6.45	6	4.8	
9	2	1.32	3	2.38	8	2.69	0	0	3	2.4	
Marital status											0.098
Married	26	17.11	31	24.6	64	21.55	17	27.42	51	40.8	
Widowed	43	28.29	26	20.63	67	22.56	11	17.74	19	15.2	
Separated	2	1.32	1	0.79	2	0.67	2	3.23	2	1.6	
Divorced	2	1.32	1	0.79	7	2.36	2	3.23	0	0	
Partnered/de factor	4	2.63	5	3.97	10	3.37	3	4.84	1	0.8	
Single	75	49.34	62	49.21	147	49.5	27	43.55	52	41.6	

*Note:* Superscripts (e.g., a, b, c, d) indicate significant pairwise comparisons for BMI. Gender, sexual orientation, education, ethnicity, and marital status are reported as counts (*n*) and percentages (%).

Abbreviations: % = percentage of participants, AN = Anorexia Nervosa, BMI = body mass index, ES = effect size, GAD = Generalized Anxiety Disorder, M = mean, MDD = Major Depressive Disorder, *n* = number of participants, *p* = *p*‐value, PD = Panic Disorder, SAD = Social Anxiety Disorder, SD = standard deviation.

**TABLE 2:** Comorbidity between diagnostic groups.

	MDD	AN	GAD	SAD	PD	Other
AN (137)	35	137	41	37	15	40
	25.55%	100.00%	29.93%	27.01%	10.95%	29.20%
MDD (152)	152	33	109	47	32	106
	100.00%	21.71%	71.71%	30.92%	21.05%	69.74%
GAD (297)	132	44	297	63	49	164
	44.44%	14.81%	100.00%	21.21%	16.50%	55.22%
SAD (126)	55	42	64	126	25	61
	43.65%	33.33%	50.79%	100.00%	19.84%	48.41%
PD (62)	33	16	43	22	62	39
	53.23%	25.81%	69.35%	35.48%	100.00%	62.90%

*Note:* The table presents the number (*n*) and percentage of participants with a primary diagnosis who also reported a comorbid diagnosis. The percentages reflect the proportion of participants within each row's primary diagnosis category. The “Other” column represents participants who provided a free‐text diagnosis that did not fall into one of the five main categories.

Abbreviations: AN = Anorexia Nervosa, GAD = Generalized Anxiety Disorder, MDD = Major Depressive Disorder, PD = Panic Disorder, SAD = Social Anxiety Disorder.

### 2.2 Data Sample

The data was part of a larger research project, that collected data from 2019 to 2023, looking at transdiagnostic factors across several mental health conditions, multiple sclerosis, cancer, and endometriosis; the current study focused solely on the mental health disorders. Specifically, we assessed IPs in MDD, AN, GAD, SAD and PD. Other mental health disorders were assessed but excluded from the analyses due to small sample sizes, including BED (*n* = 36), OSFED (*n* = 26), Specific Phobia (*n* = 27), Separation Anxiety Disorder (*n* = 46), Agoraphobia (*n* = 23), and Premenstrual Dysphoric Disorder (*n* = 43). Ethical approval was obtained from a University in Melbourne with an Ethics Committee HREC number: 1853137.6. Informed consent was obtained from all participants prior to their involvement for use of their data and publication. Participants received an e‐gift voucher for their participation. Data security was de‐identified and stored on university password protected serves.

### 2.3 Measures

An online survey which included a number of validated measures were administered to all participants to assess various dimensions of IPs, symptom severity, and demographic and clinical information distributed through Qualtrics.

For the present study the following measures were used:

*Brief Illness Perception Questionnaire (BIPQ)* (Broadbent et al. 2006): An 8‐item measure assessing cognitive and emotional representations of illness, with items rated on an 11‐point scale from 0 to 10. Higher scores indicate more negative IPs.

*Centre for Epidemiologic Studies Depression Scale (CES‐D)* (Radloff 1977): A 20‐item measure assessing depressive symptoms experienced in the past week, scored from 0 (rarely or none of the time) to 3 (most or all of the time). Total scores range from 0 to 60, with higher scores indicating greater depressive symptoms. With a Cronbach's alpha value of *α* = 0.94 the reliability of the CES‐D was excellent.

*Eating Attitudes Test (EAT‐26)* (Garner et al. 1982): A 26‐item self‐report measure assessing symptoms and concerns characteristic of eating disorders, scored from 0 (never) to 3 (always). Total scores range from 0 to 78, with higher scores indicating more severe ED symptoms. The Cronbach alpha value for the current sample for the EAT‐26 was excellent (*α* = 0.92).

*Generalized Anxiety Disorder Assessment (GAD‐7)* (Spitzer et al. 2006): A seven‐item self‐report measure assessing the severity of GAD symptoms, scored from 0 (not at all) to 3 (nearly every day). Total scores range from 0 to 21, with higher scores indicating greater severity. The Cronbach's alpha value for the GAD‐7 was good (*α* = 0.88).

*Social Interaction Anxiety Scale (SIAS)* (Mattick and Clarke 1998): A 20‐item measure assessing fear of social interactions, a measure of SAD, scored from 0 (not at all characteristic of me) to 4 (extremely characteristic of me). Total scores range from 0 to 80, with higher scores indicating greater social anxiety. The Cronbach alpha value for the current sample for the SIAS was excellent (*α* = 0.93).

*Panic Disorder Severity Scale (PDSS)* (Shear et al. 2001): A clinician‐administered scale with seven items rated on a five‐point scale, assessing the severity of symptoms. Higher scores indicate greater severity. With a Cronbach's alpha of value of *α* = 0.88 the reliability of the PDSS was considered good.

### 2.4 Procedure

Participants were recruited through university networks, local health organizations, and affiliated mental health services. Interested individuals accessed the survey via a Qualtrics link, where they were provided with detailed study information, including the purpose, procedures, and confidentiality measures. Participants provided informed consent before proceeding.

The survey was self‐administered online and asked participants to only identify illnesses that had been diagnosed by a mental health care clinician. Upon completion, responses were securely stored on password‐protected university servers. Data quality control measures included removing incomplete responses and duplicate entries. Following survey completion, participants received an electronic gift voucher as compensation for their time and contribution.

### 2.5 Statistical Analyses

All data analysis was conducted using JASP 0.19.0 with significance set at *p* < 0.05 for all statistical models. Incomplete responses and duplicates excluded prior to analysis. This exclusion maintained the integrity of the dataset and ensured accurate interpretation of the results.

A linear regression analysis was employed to assess our first aim, observing the relationship between IPs (BIPQ scores) and clinical severity (outcome measures i.e. MDD = CES‐D) for each disorder separately. The model included IPs as the predictor variable and clinical severity as the outcome variable. Age, gender, illness duration and body mass index (BMI, for the AN group only) were included as covariates to control for demographic influences. BMI was included in the AN analysis due to it being a diagnostic criterion of AN in the DSM‐5 (American Psychiatric Association 2013; Machado et al. 2017), and because starvation (a low BMI) impacts cognition (Spettigue et al. 2025).

For our second aim, looking at the unique/shared IP dimensions across mental health conditions, a Kruskal‐Wallis test was conducted to compare the BIPQ subscales across the five diagnostic groups (MDD, AN, GAD, SAD and PD). Kruskal‐Wallis was chosen due to deviations from normality detected by the Shapiro‐Wilk test in all mental health conditions, ensuring the analysis remains valid without assuming normal distribution. Levene's test for homogeneity of variances was non‐significant across all subscales (*p* > 0.05), supporting the assumption of equal variances. Post‐hoc Dunn's tests were used to explore significant pairwise group differences, with Bonferroni corrections applied to adjust for multiple comparisons. This approach provided a robust analysis while controlling for potential Type I errors due to multiple tests.

Throughout all analyses, the assumptions of the selected statistical methods were examined. Where assumptions were breeched, appropriate non‐parametric methods were used to ensure robust conclusions. The chosen analytic approach offered both disorder‐specific insights (via regression models) and cross‐diagnostic comparisons (via Kruskal‐Wallis and post‐hoc tests), providing a comprehensive assessment of the role of IPs in mental health illness severity.

## 3 Results

### 3.1 Illness Perceptions and Their Associations With Symptom Severity

Table 3 presents the associations between IPs and symptom severity across MDD, AN, GAD, SAD and PD, controlling for age, illness duration, gender and BMI (for AN only).

**TABLE 3:** Associations between BIPQ subscales and symptom severity in mental health disorders.

	MDD		SAD		GAD		PD		AN	
	*β*	*p*	*β*	*p*	*β*	*p*	*β*	*p*	*β*	*p*
Consequences	0.179	0.203	0.252	0.099	−0.123	0.195	−0.151	0.526	0.139	0.372
Timeline	−0.012	0.912	0.058	0.634	−0.11	0.169	−0.108	0.648	−0.255	0.06
Personal control	−0.108	0.278	−0.169	0.111	−0.135	**0.044**	−0.18	0.326	0.225	**0.038**
Treatment control	−0.042	0.672	−0.11	0.301	−0.014	0.833	−0.02	0.901	−0.231	**0.041**
Identity	0.079	0.542	−0.136	0.42	0.12	0.177	0.046	0.815	0.007	0.96
Concern	−0.183	0.103	0.005	0.975	0.216	**0.008**	0.203	0.219	0.027	0.812
Coherence	−0.08	0.356	−0.056	0.573	0.022	0.721	−0.078	0.577	−0.039	0.72
Emotion	0.182	0.146	0.15	0.333	0.093	0.304	0.283	0.201	−0.093	0.523
Illness duration	0.277	**0.001**	0.069	0.48	0.219	**< 0.001**	0.254	0.093	−0.163	0.136
Age	−0.259	**0.004**	−0.266	**0.004**	−0.291	**< 0.001**	−0.057	0.721	−0.11	0.288
Gender (female)	2.035	**0.044**	0.827	0.410	1.181	0.239	−0.419	0.677	1.050	0.296
BMI									−0.003	0.975

*Note: β* = Standardized regression coefficient, *p* = significance level. Symptom severity was measured using the following instruments: AN = Anorexia Nervosa (Eating Attitudes Test [EAT‐26]); MDD = Major Depressive Disorder (Centre for Epidemiologic Studies Depression Scale [CES‐D]); GAD = Generalized Anxiety Disorder (Generalized Anxiety Disorder Assessment [GAD‐7]); SAD = Social Anxiety Disorder (Social Interaction Anxiety Scale [SIAS]); PD = Panic Disorder (Panic Disorder Severity Scale [PDSS]). Bold indicates a *p* value of ≤ 0.05.

For MDD, no IP subscales were significantly related to CES‐D scores; however, longer illness duration predicted greater severity, while older age was associated with lower severity.

In AN, higher Treatment Control was linked to lower EAT‐26 scores, whereas higher Personal Control was linked to greater severity.

For GAD, higher Personal Control was associated with lower GAD‐7 scores, while greater Concern and longer illness duration were linked to higher severity; older age was associated with lower severity.

For SAD, older age predicted lower SIAS scores, with no significant associations for IP subscales.

Finally, for PD, no significant associations were observed between IPs, age, illness duration, and PDDS scores.

### 3.2 Comparing Illness Perceptions Across Mental Health Conditions

Table 4 shows the differences between IP dimensions and the five mental health disorders. Significant effects were observed for Timeline, Identity, Coherence, and Emotional Representations. Dunn's post‐hoc comparisons (Table S1), adjusted for multiple comparisons, indicated that significant differences were observed between mental health conditions across multiple subscales. On the Timeline subscale, AN showed significantly lower scores compared to all other conditions, with both GAD and MDD groups scoring significantly higher than SAD. For Identity, the AN group scored significantly lower than GAD, MDD and PD. On the Emotional Representation subscale, the AN group demonstrated significantly lower scores compared to GAD and MDD. Finally, although Coherence showed a significant overall group difference, post‐hoc comparisons did not reveal any statistically significant differences between the individual disorders.

**TABLE 4:** Kruskal‐Wallis test results for IP subscales across the psychiatric disorders.

Subscale	Statistic	Df	*P*
Consequences	6.299	4	0.178
Timeline	48.798	4	**<** **0.001**
Personal control	5.853	4	0.21
Treatment control	3.219	4	0.522
Identity	19.646	4	**<** **0.001**
Concern	7.929	4	0.094
Coherence	10.701	4	**0.03**
Emotional representations	13.449	4	**0.009**

*Note:* df = degrees of freedom, *p* = *p*‐value, *Statistic* = Kruskal‐Wallis H value. Bold indicates a *p* value of ≤ 0.05.

## 4 Discussion

The aims of the current study were twofold: (1) to investigate whether IPs were linked to symptom severity in MDD, AN, GAD, SAD and PD and (2) to explore differences and similarities in IPs across these mental disorders. With our first hypothesis we found that IPs were significantly associated with symptom severity only in AN and GAD. Specifically, we found that Personal Control was linked to reduced severity in GAD but increased severity in AN; Treatment Control was negatively associated with severity in AN; and perceived Concern was positively associated with severity in GAD. Consistent with our second hypothesis, our findings supported a largely transdiagnostic pattern; however, important differences also emerged across conditions in Timeline, Identity, and Emotional Representations, with these differences being most pronounced in AN.

### 4.1 Perceived Personal Control and Treatment Control in Anorexia Nervosa

In AN, our study identified significant associations between Personal Control and Treatment Control and illness severity, consistent with previous research highlighting the role of perceived control in ED severity (Gorwood et al. 2019). Specifically, our results indicate that individuals with AN who perceived higher levels of Personal Control over their condition reported greater eating disorder severity. This suggests that in AN, perceived Personal Control may be maladaptive, reinforcing rigid control over food intake, body weight, and other disordered behaviours that exacerbate the illness (Agüera et al. 2021; Haynos et al. 2018; Pauligk et al. 2021).

Our findings also demonstrated that for the AN sample higher perceptions of Treatment Control were associated with lower eating disorder symptom severity, suggesting that a belief in the efficacy of treatment maybe a protective factor. This aligns with a growing body of literature demonstrating that strong beliefs in the controllability of a condition through treatment often lead to improved adherence, higher treatment expectations, and better clinical outcomes across a range of health issues (von der Warth et al. 2022; Chan and Mak 2016).

It is important to note that many individuals with AN also experienced comorbid anxiety or depression, which may have shaped the relationship between perceived control and illness severity. Elevated Personal Control may reflect not only eating‐specific cognitions but also a broader tendency toward rigidity and perfectionism compounded by comorbid anxiety. Conversely, beliefs in Treatment Control might be weakened in the presence of comorbid depression, where hopelessness can undermine optimism about treatment efficacy.

While comorbidity may have influenced these perceptions, our findings nonetheless highlight the importance of considering Personal Control and Treatment Control within the AN individuals. Our findings suggest that both Personal Control and Treatment Control could be important targets for recovery in AN. Helping individuals with AN develop a more flexible and adaptive sense of control, focused on healthy behaviours rather than rigidity in their disordered eating patterns (Robertson and Thornton 2021) and greater appreciation for Treatment Control, may reduce the severity of eating disorder symptoms in AN.

### 4.2 Concern and Personal Control Shape Anxiety Severity in Generalised Anxiety Disorder

In our study, a greater perceived sense of Personal Control was associated with lower anxiety severity, while a higher level of Concern was related to increased anxiety symptoms. Ou findings align with previous research, such as the study by Yap et al. (2019), which found that negative perceptions of Personal Control and the perceived Consequences of anxiety significantly contribute to heightened anxiety levels. This may reflect how emotional distress may heighten both the mental and physical strain of anxiety, where intense emotional interpretations and preoccupation can lead individuals to become overly sensitive to perceived threats (Bystritsky and Kronemyer 2014; Hirsch et al. 2015).

Given the high rates of comorbidity between GAD and depression (Groen et al. 2020; Ter Meulen et al. 2021), it is possible that overlapping symptoms (e.g., fatigue, concentration problems) or shared cognitive vulnerabilities influenced our findings. For instance, the positive association between Concern and anxiety severity may partly reflect ruminative processes common to both GAD and depression (Baik and Newman 2025), making it difficult to disentangle disorder‐specific IPs from transdiagnostic features driven by comorbidity.

Furthermore, extended duration of illness and advancing age were both correlated with higher GAD‐7 scores, which reflects our current understanding of a longer duration of untreated illness leading to poorer response to treatment and worse outcomes (Altamura et al. 2008).

Fostering a sense of personal control may empower GAD patients to manage their anxiety more effectively, reducing its overall impact. This reflects the goals of most psychotherapy approaches to anxiety, which aim to provide people with skills to manage their distress and cognitions (Papola et al. 2024; Nakao et al. 2021). Conversely, our finding that higher levels of Concern were associated with increased GAD symptom severity suggests that addressing excessive worry about the condition itself may be a crucial therapeutic target.

### 4.3 Age as a Predictor of Social Anxiety Severity

Our study revealed no significant associations between IPs and clinical severity of individuals with SAD. However, we did find that age was significantly and negatively associated with SAD severity. The inverse association between age and SAD severity may reflect the developmental trajectory of the condition, with a growing prevalence of SAD among younger populations (Jefferies and Ungar 2020).

While our study did not find a direct link between IPs and illness severity in SAD, the literature highlights the importance of perceived control and consequences in managing this diagnosis (Dias et al. 2018; Hur et al. 2020). Previous research suggests that interventions such as exposure therapy and cognitive restructuring may help shift perceptions of consequences, allowing individuals to more actively engage in social situations and develop coping mechanisms (Butler et al. 2021; Newman et al. 2023).

### 4.4 Illness Perceptions in Panic Disorder Do Not Impact Clinical Severity

Building on the work of Dias et al. (2018), who demonstrated that PD patients exhibited significantly higher anxiety sensitivity and lower perceived personal and treatment control compared to SAD patients, our study offers additional perspectives with a larger and more diverse sample. However, in contrast to Dias et al.'s findings, our data did not reveal statistically significant relationships between IPs and PD severity. This discrepancy suggests that while PD patients may perceive their condition as having significant negative consequences and a chronic timeline, these perceptions do not necessarily correlate with the severity of their panic symptoms.

Several factors may help explain the discrepancy between our findings and those of Dias et al. (2018). One notable difference lies in the sample sizes: our study included 62 individuals with PD, compared to 36 participants in Dias et al.’s study. While our sample is larger and somewhat more diverse, it is still relatively modest in size, which may limit the strength and generalizability of the conclusions. Future studies with larger, multi‐site cohorts will be important to clarify these differences and strengthen the evidence base.

Overall, the lack of significant findings for PD and SAD may partly reflect their frequent comorbidity with depression and other anxiety disorders (Kalin 2020). Comorbid depression, for example, could overshadow disorder‐specific IPs, with depressive cognitions about control or consequences diluting associations within these groups. This heterogeneity may have reduced the likelihood of detecting disorder‐specific IP–severity links.

### 4.5 Illness Duration and Gender as a Key Driver of Clinical Severity in Major Depressive Disorder

Our findings for MDD did not reveal any significant associations between IPs and clinical severity. However, we found that longer illness duration and female gender were associated with greater depressive symptom severity. While the link between illness duration and chronicity aligns with prior research (Monroe and Harkness 2022; Ten Have et al. 2018), our finding of gender differences contrasts with literature that often reports no significant differences (Parker et al. 2014).

Our findings highlight the need to consider illness duration in MDD interventions, particularly for long‐standing cases where managing chronic symptoms and preventing recurrence is essential. Although our study did not find specific IPs to be significant, therapies such as Cognitive Behavioural Therapy (Beck 1970) remain important for targeting cognitive distortions and reducing symptom burden.

### 4.6 Comparison of Illness Perceptions Across Mental Health Disorders

Supporting our second hypothesis, we found that most IP subscales showed transdiagnostic features, with a few disorders displaying unique profiles. AN was particularly distinct from other mental health conditions on Timeline, Identity, and Emotional Representations.

When looking at how individuals perceive their illness identity, individuals with MDD often viewed their illness as a more integral part of their identity compared to those with AN. This suggests that MDD may be perceived as a core aspect of the self, potentially due to its pervasive impact on mood and daily functioning (Kendler 2016), often leading to a profound alteration in self‐concept and daily life. The chronic and relapsing nature of depressive symptoms can deeply affect an individual's sense of self, reinforcing the perception that the illness is an inseparable part of their identity (Kessler and Bromet 2013; Manber et al. 2003; Kendler 2016).

In contrast, individuals with AN frequently perceive themselves as having greater control over their condition. This perception is a critical component of their clinical profile, where a sense of control is often overvalued (Gorwood et al. 2019; Agüera et al. 2021; Holliday et al. 2005; Fairburn et al. 1999). It has been suggested that this belief can manifest as a defence mechanism against the perceived chaos or lack of control in other areas of life, or as part of the cognitive distortions characteristic of the disorder (Fairburn et al. 1999; Brewerton and Dennis 2016). The strong focus on control overeating and body image, despite serious health consequences, illustrates a poor insight into the severity and complexity of their condition (Gorwood et al. 2019; Arbel et al. 2013; Vandereycken 2006a, 2006b).

Regarding perceptions of illness duration, individuals with anxiety disorders generally perceived their condition as more chronic compared to those with AN. This is consistent with the clinical profile of anxiety disorders, where symptoms such as persistent worry and hypervigilance create a continuous state of mental and physical tension (Angst and Vollrath 1991; Hovenkamp‐Hermelink et al. 2021). The persistent nature of anxiety symptoms, often exacerbated by triggers and stressors, contrasts with the perception in AN, where a greater sense of control might lead to underestimating the disorder's chronicity. These cross‐disorder comparisons demonstrate the complexity and diversity of IPs, reinforcing why a one‐size‐fits‐all approach is unlikely to be effective in clinical practice.

When interpreting these cross‐disorder comparisons, it is also crucial to acknowledge that comorbidity likely influenced group differences. For example, individuals with AN and comorbid anxiety may report higher Concern or Emotional Representations compared to those with ‘pure’ AN, making AN appear more distinct. Similarly, the degree to which MDD was perceived as part of identity may be intensified when occurring alongside long‐standing anxiety disorders. Thus, what appear to be disorder‐specific IPs may in part reflect comorbid clinical features.

### 4.7 Implications of Findings

Our results have important implications for understanding and addressing IPs in clinical practice. Tailoring treatment interventions to target maladaptive IPs, such as perceptions of illness chronicity or lack of personal control, may enhance treatment engagement and improve outcomes for individuals with mental health disorders. Additionally, our findings underscore the importance of considering individual differences in IPs when developing personalized treatment approaches.

Given the high prevalence of comorbidity across the disorders examined, interventions may also need to address overlapping or compounded IPs that span multiple conditions. For instance, comorbid anxiety and depression may intensify maladaptive beliefs about uncontrollability or chronicity, while co‐occurring eating and anxiety disorders could reinforce rigid perceptions of personal control. Recognising and targeting these transdiagnostic patterns within comorbid presentations may therefore be critical for optimizing treatment outcomes.

Our study's findings are particularly relevant to the ongoing shift towards the recovery model in psychiatry (Davidson et al. 2021), which emphasizes patient‐centred care and greater involvement of patients in their own treatment and decision‐making processes. The recovery model advocates for a holistic approach that considers the individual's subjective experience of their illness, aiming to empower patients by involving them actively in their treatment plans (Leamy et al. 2011; Slade et al. 2008). By elucidating the significant relationships between IPs and clinical severity across various mental health disorders, our research highlights the importance of understanding and addressing these perceptions in clinical practice. Tailoring interventions to align with patients' views and experiences can enhance engagement and adherence to treatment, fostering a sense of ownership and control over their recovery journey (Leamy et al. 2011). This approach aligns with the recovery model's principles of personal empowerment and collaborative care, promoting not just symptom reduction but also the achievement of a meaningful and fulfilling life despite the presence of mental illness (Anthony 1993). Therefore, incorporating IP assessments into routine clinical practice could significantly enhance the efficacy of mental health care and support the broader goals of the recovery model.

### 4.8 Limitations, Strengths and Future Directions

This study has several important limitations. First, the cross‐sectional design precludes causal inferences, making it unclear whether IPs shape clinical severity or vice versa. Future research should employ longitudinal or experimental designs to clarify temporal and causal relationships.

Second, all data relied on self‐report measures, introducing potential recall bias, social desirability effects, and item misinterpretation. Future work could combine self‐report with clinician‐rated assessments or objective behavioural/biological measures to improve validity.

Third, diagnoses were self‐reported, based on prior clinical diagnoses rather than structured clinical interviews, which may affect diagnostic accuracy and overlook comorbidities or illness chronicity. Subsequent studies should incorporate standardized diagnostic interviews to enhance diagnostic precision and capture comorbid patterns.

Fourth, although we controlled for illness duration, gender, and with BMI in AN, we did not account for other clinical variables such as treatment status or functional impairment, which could influence both IPs and symptom severity. Future research should integrate these variables to provide a more comprehensive understanding of these relationships.

Fifth, we did not systematically assess comorbid conditions. Given the high rates of co‐occurrence among the disorders studied, comorbidity may have significantly influenced both IPs and severity, potentially obscuring or inflating associations. Future studies should incorporate structured diagnostic interviews to assess and control for comorbidity, and examine whether certain disorder combinations (e.g., AN + GAD, MDD + SAD) are associated with distinct IP constellations.

Sixth, recruitment from university settings and health organizations may limit generalizability, as participants may not represent more diverse cultural, geographical, or socioeconomic backgrounds. Broader recruitment strategies, including community‐based and cross‐cultural samples, are needed to enhance representativeness and external validity.

Finally, the study's strengths should also be acknowledged, which include its cross‐diagnostic examination of IPs across multiple mental health disorders, the use of validated measures, and a large sample enabling robust analyses. By focussing on IPs ‐ a factor often overlooked in mental health research ‐ it offers valuable insights to guide recovery‐oriented, patient‐centred interventions.

## 5 Conclusion

In conclusion, our study underscores the pivotal role of IPs in shaping the clinical severity and treatment outcomes of various mental health disorders. By examining IPs in MDD, AN, GAD, SAD and PD concurrently, we have highlighted patterns and differences that would remain undiscovered in single‐disorder studies, thereby offering a more comprehensive understanding of how IPs intersect with symptom severity. These findings align with the principles of the recovery model, which advocates for empowering patients through active involvement in their treatment plans and acknowledging their subjective experiences. Nevertheless, findings should be interpreted with caution given the high levels of comorbidity across the mental disorders assessed. Addressing maladaptive IPs, may enhance therapeutic engagement and effectiveness, ultimately fostering a more holistic and person‐centred approach to mental health care.

## Ethics Statement

Ethical approval was obtained from the University of Melbourne with an Ethics Committee HREC number: 1853137.6.

## Consent

Informed consent was obtained from all participants prior to their involvement for use of their data and publication.

## Conflicts of Interest

The authors declare no conflicts of interest.

## Supporting information


**Table S1:** Dunn’s Post‐Hoc Comparisons Across All Significant Subscales Between Mental Health Conditions.

## Data Availability

The datasets used and analysed during the current study are available from the corresponding senior authors on reasonable request, subject to ethical and privacy considerations.
