# Supplementary material for: Illness Perceptions in Psychiatric Disorders: Assessing Differences and Associations With Symptom Severity
Source: Eur Eat Disord Rev. 2025 Oct 8;34(2):398–410. doi: 10.1002/erv.70036 (PMC12862552; doi:10.1002/erv.70036)
Supplement: Supplementary file 1 — Table S1: Dunn’s Post‐Hoc Comparisons Across All Significant Subscales Between Mental Health Conditions. [file ERV-34-398-s001.docx]

Supplementary tables

**Table S1**

Dunn’s Post-Hoc Comparisons Across All Significant Subscales Between Mental Health Conditions

|  | **Dunn’s Post-Hoc Comparisons** | | | | | | |
| --- | --- | --- | --- | --- | --- | --- | --- |
| Subscale | Comparison | *z* | *Wi* | *Wj* | *p* | *pbonf* | *pholm* |
| Timeline | AN - GAD | -6.204 | 274.332 | 414.283 | < 0.001 | < 0.001 | < 0.001 |
|  | AN - MDD | -5.656 | 274.332 | 418.842 | < 0.001 | < 0.001 | < 0.001 |
|  | AN - PD | -4.336 | 274.332 | 416.863 | < 0.001 | < 0.001 | < 0.001 |
|  | AN - SAD | -2.761 | 274.332 | 348.095 | 0.006 | 0.058 | 0.033 |
|  | GAD - MDD | -0.216 | 414.283 | 418.842 | 0.829 | 1 | 1 |
|  | GAD - PD | -0.087 | 414.283 | 416.863 | 0.93 | 1 | 1 |
|  | GAD - SAD | 2.942 | 414.283 | 348.095 | 0.003 | 0.033 | 0.023 |
|  | MDD - PD | 0.062 | 418.842 | 416.863 | 0.951 | 1 | 1 |
|  | MDD - SAD | 2.775 | 418.842 | 348.095 | 0.006 | 0.055 | 0.033 |
|  | PD - SAD | 2.095 | 416.863 | 348.095 | 0.036 | 0.362 | 0.145 |
| Identity | AN - GAD | -3.041 | 314.476 | 385.322 | 0.002 | 0.024 | 0.019 |
|  | AN - MDD | -3.864 | 314.476 | 416.434 | < 0.001 | 0.001 | 0.001 |
|  | AN - PD | -3.529 | 314.476 | 434.274 | < 0.001 | 0.004 | 0.004 |
|  | AN - SAD | -2.044 | 314.476 | 370.873 | 0.041 | 0.409 | 0.287 |
|  | GAD - MDD | -1.428 | 385.322 | 416.434 | 0.153 | 1 | 0.46 |
|  | GAD - PD | -1.604 | 385.322 | 434.274 | 0.109 | 1 | 0.435 |
|  | GAD - SAD | 0.622 | 385.322 | 370.873 | 0.534 | 1 | 1 |
|  | MDD - PD | -0.542 | 416.434 | 434.274 | 0.588 | 1 | 1 |
|  | MDD - SAD | 1.73 | 416.434 | 370.873 | 0.084 | 0.836 | 0.418 |
|  | PD - SAD | 1.87 | 434.274 | 370.873 | 0.061 | 0.615 | 0.369 |
| Coherence | AN - GAD | -2.287 | 339.852 | 393.111 | 0.022 | 0.222 | 0.2 |
|  | AN - MDD | -2.064 | 339.852 | 394.283 | 0.039 | 0.391 | 0.273 |
|  | AN - PD | -2.664 | 339.852 | 430.25 | 0.008 | 0.077 | 0.077 |
|  | AN - SAD | -0.587 | 339.852 | 356.04 | 0.557 | 1 | 1 |
|  | GAD - MDD | -0.054 | 393.111 | 394.283 | 0.957 | 1 | 1 |
|  | GAD - PD | -1.218 | 393.111 | 430.25 | 0.223 | 1 | 0.894 |
|  | GAD - SAD | 1.596 | 393.111 | 356.04 | 0.11 | 1 | 0.663 |
|  | MDD - PD | -1.093 | 394.283 | 430.25 | 0.275 | 1 | 0.894 |
|  | MDD - SAD | 1.453 | 394.283 | 356.04 | 0.146 | 1 | 0.731 |
|  | PD - SAD | 2.19 | 430.25 | 356.04 | 0.029 | 0.285 | 0.228 |
| Emotional Representation | AN - GAD | -2.778 | 322.428 | 387.049 | 0.005 | 0.055 | 0.049 |
|  | AN - MDD | -3.468 | 322.428 | 413.773 | < 0.001 | 0.005 | 0.005 |
|  | AN - PD | -2.428 | 322.428 | 404.71 | 0.015 | 0.152 | 0.121 |
|  | AN - SAD | -1.97 | 322.428 | 376.671 | 0.049 | 0.489 | 0.342 |
|  | GAD - MDD | -1.228 | 387.049 | 413.773 | 0.219 | 1 | 1 |
|  | GAD - PD | -0.58 | 387.049 | 404.71 | 0.562 | 1 | 1 |
|  | GAD - SAD | 0.447 | 387.049 | 376.671 | 0.655 | 1 | 1 |
|  | MDD - PD | 0.276 | 413.773 | 404.71 | 0.783 | 1 | 1 |
|  | MDD - SAD | 1.412 | 413.773 | 376.671 | 0.158 | 1 | 0.948 |
|  | PD - SAD | 0.829 | 404.71 | 376.671 | 0.407 | 1 | 1 |

**Note**. z = standardized test statistic; Wi = sum of ranks for group i; Wj = sum of ranks for group j; p = uncorrected p-value; pbonf = Bonferroni-adjusted p-value; pholm = Holm-adjusted p-value. AN = Anorexia Nervosa; MDD = Major Depressive Disorder; GAD = Generalized Anxiety Disorder; SAD = Social Anxiety Disorder; PD = Panic Disorder.
